# Treatment outcomes and prognostic indicators of primary immune thrombocytopenia in 31 cats: a multicenter retrospective study (2000-2023)

**DOI:** 10.1093/jvimsj/aalaf038

**Published:** 2026-01-21

**Authors:** Mulan Zhong, Evelyn Hall, Barbara Glanemann, Susan H S Jih, Rachel M Korman, Rebecca Langhorn, Jana Leshinsky, Amy E Lingard, Elodie Roels, Martine van Boeijen, Lara A Boland

**Affiliations:** Sydney School of Veterinary Science, Faculty of Science, The University of Sydney, Sydney, New South Wales 2006, Australia; Sydney School of Veterinary Science, Faculty of Science, The University of Sydney, Sydney, New South Wales 2006, Australia; Queen Mother Hospital for Animals, The Royal Veterinary College, Hatfield, Hertfordshire, United Kingdom; The Cat Clinic, Brisbane, Queensland, Australia; Veterinary Specialist Services, Brisbane, Queensland, Australia; Department of Veterinary Clinical Sciences, University of Copenhagen, Frederiksberg, Denmark; Small Animal Specialist Hospital, Ryde, New South Wales, Australia; The Cat Doctors, Melbourne, Victoria, Australia; Department of Clinical Sciences, FARAH, Faculty of Veterinary Medicine, University of Liège, Liège, Belgium; Perth Cat Hospital, Perth, Western Australia, Australia; Sydney School of Veterinary Science, Faculty of Science, The University of Sydney, Sydney, New South Wales 2006, Australia; Small Animal Specialist Hospital, Ryde, New South Wales, Australia

**Keywords:** autoimmune, platelet, thrombocytopenic, feline

## Abstract

**Background:**

Primary immune thrombocytopenia (ITP) in cats is rare. Evidence is lacking on effective immunosuppressive protocols, prognosis, and prognostic indicators.

**Hypothesis/Objectives:**

To investigate immunosuppressive treatment outcomes and prognostic indicators of cats with primary ITP.

**Animals:**

Thirty-one cats with primary ITP.

**Methods:**

Multi-institutional retrospective cohort study (2000-2023). Cats were identified by searching medical records from 12 institutions. Immunosuppressive treatments and prognostic indicators were studied in association with clinical outcomes. Indicators included age, sex, breed, bodyweight, initial platelet count and PCV, blood urea nitrogen concentration, and blood product administration. Outcomes including medical control (platelet count > 57 000/μL), relapse, remission, death, and survival to discharge were analyzed using logistic regression models. Hospitalization duration, days to medical control, remission, and death were assessed using linear regression models. Survival was assessed using the Kaplan–Meier method.

**Results:**

Of the cats included, 27/31 (87%) survived to discharge, 25/31 (81%) achieved medical control, 8/25 (32%) achieved remission, 10/25 (40%) relapsed during immunosuppressive tapering, and 5/10 (50%) had a second relapse. Cats administered corticosteroids and cyclosporine had longer hospitalizations than corticosteroids alone (8.4 ± 0.9 vs 4.4 ± 0.9 days; *P* = .005). Median survival was not reached. Median follow-up was 315 days (95% CI, 216-1252).

**Conclusions and clinical importance:**

Cats with primary ITP have a fair short-term prognosis and low remission rate. The frequent relapse rate warrants close monitoring. Besides longer hospitalization durations, combination immunosuppressive treatment did not affect other outcomes, when compared to corticosteroids alone.

## Introduction

Primary, nonassociative, immune thrombocytopenia (ITP) is a rare cause of thrombocytopenia in cats.^1^^,^^2^ The autoimmune disorder is proposed to elicit autoantibody and cytotoxic T cell-mediated platelet destruction, and to impair thrombopoiesis.^3^ Primary ITP is idiopathic and diagnosed by excluding secondary (associative) causes, such as infectious diseases (eg, feline immunodeficiency virus [FIV] infection, feline leukemia virus [FeLV]) infection, inflammatory diseases (eg, pancreatitis), malignant neoplasia, and drugs.^4^ Immune thrombocytopenia can occur in combination with immune-mediated hemolytic anemia, a complex also known as Evans syndrome, although this is rarely reported in cats.^1^^,^^5^ Few case reports and series are reported of cats with primary ITP, and there is weak evidence supporting consensus on treatment.^6–11^ Little is known about prognostic indicators and prognosis.

First-line medical management for primary ITP commonly involves administration of corticosteroids, with prednisolone administered at 2-4 mg/kg/day.^6–9^ In previous reports, 2 cats refractory to prednisolone responded when administered dexamethasone instead.^7^^,^^9^ Second-line immunosuppressives might be considered if there is a poor response to corticosteroids, with the presence of severe or refractory hemorrhage, or in response to a relapse while tapering the first agent.^11^ There is a lack of knowledge on the efficacy of combination immunosuppressive treatment and weak evidence-based preference for second-line immunosuppressants. Recommended second-line immunosuppressants include cyclosporine and chlorambucil.^11^ No case reports describe cats being weaned off immunosuppressants successfully.^6–10^ In dogs with primary ITP, there is no association between different immunosuppressive protocols and survival to discharge or relapse.^12^ The survival to discharge rate in dogs ranges from 84% to 97%, and the relapse rate from 9% to 47%.^13–18^ Negative prognostic indicators in dogs with ITP include melena, a lower PCV, and above reference range blood urea nitrogen (BUN) concentration.^14^^,^^19^^,^^20^ Prognostic indicators are not described in cats.

The aim of the present study was to investigate the response to immunosuppressive protocols and prognostic indicators of cats with primary ITP. The objectives were as follows: (1) to retrospectively review the clinicopathological features and clinical outcomes of cats with primary ITP, (2) to compare the efficacy of different immunosuppressive protocols on the clinical outcome of cats with primary ITP, and (3) to identify prognostic indicators in cats with primary ITP.

## Material and methods

### Case selection

Veterinary hospitals were invited to participate in this retrospective cohort study to identify cats diagnosed with primary ITP between January 2000 and March 2023. Ethical approval for data collection in Denmark was provided by the Ethical and Administrative Committee at the Department of Veterinary Clinical Sciences, University of Copenhagen (reference number 2023-31). At other institutions, ethical approval was not required because of the retrospective and de-identified nature of the study. Potential cats were filtered from each hospital’s database through either search terms, medical records, or laboratory reports. Search terms included “IMT,” “IMTP,” “IM thrombocytopenia,” “Immune mediated thrombocytopenia,” “Autoimmune thrombocytopenia,” and “Primary thrombocytopenia.” A nominated veterinarian from each hospital provided de-identified details of these cats using a spreadsheet. Details collected included signalment, history, clinical signs, physical examination findings, diagnostic test results, treatment, and clinical outcomes.

All cats were reviewed against the study’s eligibility criteria by 2 authors (M.Z./L.B.). The inclusion criteria comprised cats with platelet counts below 100 000/μL in accordance with the American College of Veterinary Internal Medicine consensus statement guidelines, a full physical examination, and the following minimum diagnostic tests: thoracic and abdominal imaging (radiography, ultrasonography, computed tomography, or a combination), complete blood count with blood smear examination, serum biochemistry, and infectious disease tests as appropriate for the geographical region.^21^ A minimum of 30 days of follow-up was required unless the cat was euthanized or died before this timeframe. The exclusion criteria comprised secondary ITP or other potential causes of thrombocytopenia, cats with Evans syndrome, less than minimum diagnostic testing, or a partial medical record.

### Measures of clinical outcome

The following clinical outcomes were defined: medical control, relapse, remission, and recurrence. “Medical control” was defined as reaching an adequate platelet count (>57 000/μL) to minimize spontaneous bleeding, extrapolated from a study that observed spontaneous bleeding in cats below this threshold.^4^ A “relapse” was defined as a reduction in platelet count (<57 000/μL) while tapering immunosuppressants in a cat that had achieved medical control. “Remission” was defined as an absence of clinical and clinicopathologic signs of primary ITP once the cat was weaned off immunosuppressants. For cats that achieved remission, “recurrence” was defined as the return of clinical and clinicopathological signs of primary ITP.

Survival was categorized as: death associated with primary ITP, death not associated with primary ITP, euthanasia associated with primary ITP, and euthanasia not associated with primary ITP. For each of the above clinical outcomes, days from the start of immunosuppressive treatment were recorded. Duration of hospitalization was recorded as days from hospital admission to discharge.

### Statistical analyses

Statistical analyses were conducted with Genstat 22nd edition (VSN International Ltd, Hemel), and RStudio (version December 1, 2023). Immunosuppressive treatments and possible prognostic indicators were evaluated against clinical outcomes. Variables were assessed for normality using the Shapiro–Wilks test, with any variables not meeting the assumption log_e_-transformed for analysis. Corticosteroids as a single agent was compared to combination treatment of either corticosteroids with any other immunosuppressive drugs or with cyclosporine. Potential prognostic indicators evaluated included age, sex (female or male), breed (pedigree or non-pedigree), initial bodyweight, initial platelet count, initial PCV, BUN concentration above the reference range, and treatment with a blood transfusion. Age, initial bodyweight, initial platelet count, and initial PCV were assessed as continuous variables. Domestic short and long hairs, along with crossbreeds were classified under the term “non-pedigree.” All other cats were pedigrees. Univariable logistic regression models were used to estimate the odds ratios and 95% confidence intervals of the following binary outcomes: occurrence of medical control, relapse, remission, death associated with primary ITP, and survival to discharge. Univariable linear regression models were used to evaluate continuous outcomes, including days to medical control from the start of immunosuppressive treatment, days to remission after reaching medical control, and hospitalization duration. Survival was assessed using a Kaplan–Meier survival curve. Cases were right-censored at the point of death not associated with primary ITP, euthanasia not associated with primary ITP, or when lost to follow-up. A reverse Kaplan–Meier survival curve was used to assess median follow-up time. Statistical significance was defined as *P* value < .05.

## Results

### Cats

**Figure 1:**
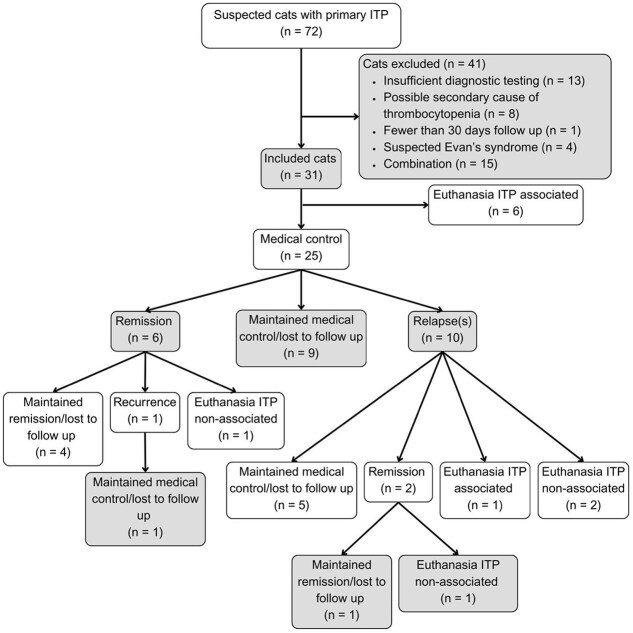
Flow diagram illustrating the inclusion of cats and their clinical outcomes. Abbreviation: ITP = immune thrombocytopenia.

A total of 72 cats were identified from 12 veterinary hospital databases (Figure 1). Thirty-one cats from 8 veterinary hospitals were included and 41 were excluded for reasons outlined in Figure 1. The geographical distribution of included cats was as follows: Australia (*n* = 15), United Kingdom (*n* = 9), Belgium (*n* = 4), and Denmark (*n* = 3). The median age was 4 years 9 months (range: 2 months-13 years 7 months). Fifteen of the cats were male (48%, 13 neutered males, 2 entire males) and 16 were female (52%, 13 neutered females, 3 entire females). Breeds included both non-pedigree (42%, *n* = 13) and pedigree (58%, *n* = 18) cats. Pedigree breeds included British Shorthair (*n* = 4), Siamese (*n* = 2), Devon Rex (*n* = 2), Persian (*n* = 2), Ragdoll (*n* = 2), and 6 cats of other breeds. The median initial bodyweight was 4.11 kg (range: 1.50-9.15 kg; missing data = 1).

Common clinical signs and physical examination findings included pallor (*n* = 24), petechiae (*n* = 20), heart murmur (*n* = 15), tachypnea (*n* = 15), inappetence (*n* = 13), ecchymosis (*n* = 13), lethargy (*n* = 12), tachycardia (*n* = 10), oral cavity bleeding (*n* = 9), vomiting (*n* = 8), epistaxis (*n* = 7), a gallop rhythm (*n* = 4), and melena (*n* = 3). Petechiae were commonly reported on the ears (*n* = 9), oral cavity (*n* = 8), and abdomen (*n* = 7). Ecchymoses were commonly reported on the abdomen (*n* = 6) and ears (*n* = 4). All 31 cats were thrombocytopenic initially with a median platelet count of 7000/μL (range: 0-40 000/μL) and had a median PCV of 14% (range: 5%-33%; missing data = 4). Six cats had a serum BUN concentration above the reference range at presentation. Prothrombin time (*n* = 18) and activated partial thromboplastin time (*n* = 16) were measured, with mild prolongation of the former in 1 cat and the latter in 2 cats. All cats had negative testing for retrovirus infection. This included point-of-care (POC) FIV antibody and FeLV antigen tests (*n* = 29), and PCR on blood (*n* = 1) or bone marrow (*n* = 1). The other 4 had a combination of POC testing with FeLV PCR on blood (*n* = 2), FeLV PCR on bone marrow (*n* = 2), or FeLV immunofluorescence assay on bone marrow (*n* = 1). One tested faintly positive on POC testing for FeLV; however, was negative on PCR. A Coombs test was performed in 6 cats and was positive in 2 cats. Saline agglutination testing was conducted in 18 cats, of which one was positive. The cats that tested positive with the Coombs test were negative on saline agglutination testing. These cats only had one marker of immune-mediated destruction and did not display signs of hemolysis.

Two cats had both a bone marrow cytology and biopsy report available. One cat had marked megakaryocytic, erythroid, and myeloid hyperplasia on both cytology and biopsy. The other cat had megakaryocytes on cytology which were not prevalent enough to judge platelet production, but had possible megakaryocytic hyperplasia, erythroid normoplasia, and myeloid normoplasia on biopsy. Six cats only had bone marrow cytology performed, with findings including megakaryocytic hyperplasia (*n* = 3), megakaryocytic normoplasia to hyperplasia (*n* = 1), megakaryocytic normoplasia (*n* = 1), and a cat with subjectively insufficient megakaryocytes (*n* = 1). Serum thyroxine concentration was tested in 7 cats. One cat had a total thyroxine concentration above the reference range first documented on the day of primary ITP diagnosis. Hemotropic mycoplasma PCR tests were performed in 11 cats, all of which were negative. One cat had *Anaplasma* species PCR testing which was negative. No cats were tested for platelet-bound antibodies.

### Treatment

Corticosteroids were administered as a single agent (*n* = 13) and combination immunosuppressive treatment (*n* = 18). Prednisolone was administered to 30 cats at a median dosage of 3.23 mg/kg/day (range: 1.8-6.0 mg/kg/day). Dexamethasone was initially administered intravenously to 9 cats at a median dosage of 0.4 mg/kg/day (range: 0.14-0.6 mg/kg/day), before transition to prednisolone treatment per os in 8 cats. The 1 cat that remained on dexamethasone did not survive to discharge. The predominant combination treatment was corticosteroids with cyclosporine (*n* = 12). Cyclosporine was administered at a median dosage of 6 mg/kg/day (range: 3.87-10 mg/kg/day). Where provided, the rationale for commencing a second agent was inadequate platelet or PCV response (*n* = 5), development of additional clinical signs (*n* = 1), or a combination (*n* = 2). Five cats were started on cyclosporine on the same day as corticosteroids. Otherwise, the administration of cyclosporine was commenced at a median of 5 days (range: 1-10 days) after corticosteroid treatment. Other immunosuppressive protocols were used in 6 cats; however, because of low sample size, these were not analyzed statistically. These protocols included corticosteroids and chlorambucil (*n* = 3); corticosteroids, chlorambucil, and vincristine (*n* = 2); and corticosteroids, cyclosporine, and vincristine (*n* = 1). Chlorambucil was predominantly administered at a total dosage of 2 mg every other day (*n* = 3), with 1 cat on 1 mg every other day and another cat on 2 mg every third day. A single dose of vincristine was administered to 3 cats, with one receiving a 0.5 mg/m^2^ dose (day 6 of treatment) because of persistent thrombocytopenia, the second a 0.01 mg/kg dose (day 2 of treatment) for an unspecified reason, and the third did not have a dosage reported.

Blood products were administered to 18 cats, with a median number of 1 transfusion (range: 1-4 transfusions). Cats received feline-typed whole blood (*n* = 11), packed red blood cells (*n* = 5), or a combination (*n* = 2). Of the cats that received packed red blood cells, 3 received feline packed red blood cells, 1 received xenotransfused canine packed red blood cells, and 1 was unspecified. The 2 cats that received a combination of blood products had both packed red blood cells and whole blood. One cat that was administered feline-typed whole blood also received synthetic hemoglobin (Oxyglobin). Common non-immunosuppressive medications administered included doxycycline (*n* = 17), sucralfate (*n* = 5), omeprazole (*n* = 5), vitamin K1 (*n* = 3), famotidine (*n* = 2), and furosemide (*n* = 2). Furosemide was administered to these cats because of clinical and radiographic findings suggestive of fluid overload.

### Clinical outcomes

The clinical outcomes of included cats are summarized in Figure 1. In this cohort, 25/31 (81%) achieved medical control at a median of 4.5 days (range: 2-187 days) and 27/31 (87%) survived to discharge after a median hospitalization duration of 7 days (range: 1-14 days; missing data = 1). The median platelet count at the time of medical control was 157 000/μL (range: 60 000-953 000/μL). Of the cats that reached medical control, relapse occurred in 10/25 cats (40%). The first relapse occurred at a median of 88.5 days (range: 17-582 days) after achieving medical control while tapering immunosuppressive medications. The median platelet count at the time of first relapse was 43 000/μL (range: 8000-50 000/μL). Subsequent relapses occurred in 5/10 cats (50%), with total relapses of 2 (*n* = 2), 3 (*n* = 2), and 4 (*n* = 1). After reaching medical control, remission was achieved in 8/25 cats (32%), at a median of 10.5 months (range: 3-15 months; missing data = 2). Two cats that achieved remission had previous relapses before. The number of relapses they had before remission was 1 and 3. Of the cats that achieved remission, 1 had a recurrence of primary ITP at 35 days after discontinuation of immunosuppressants. This cat had not had a relapse before remission. Recurrence was not statistically analyzed because of sample size.

**Figure 2:**
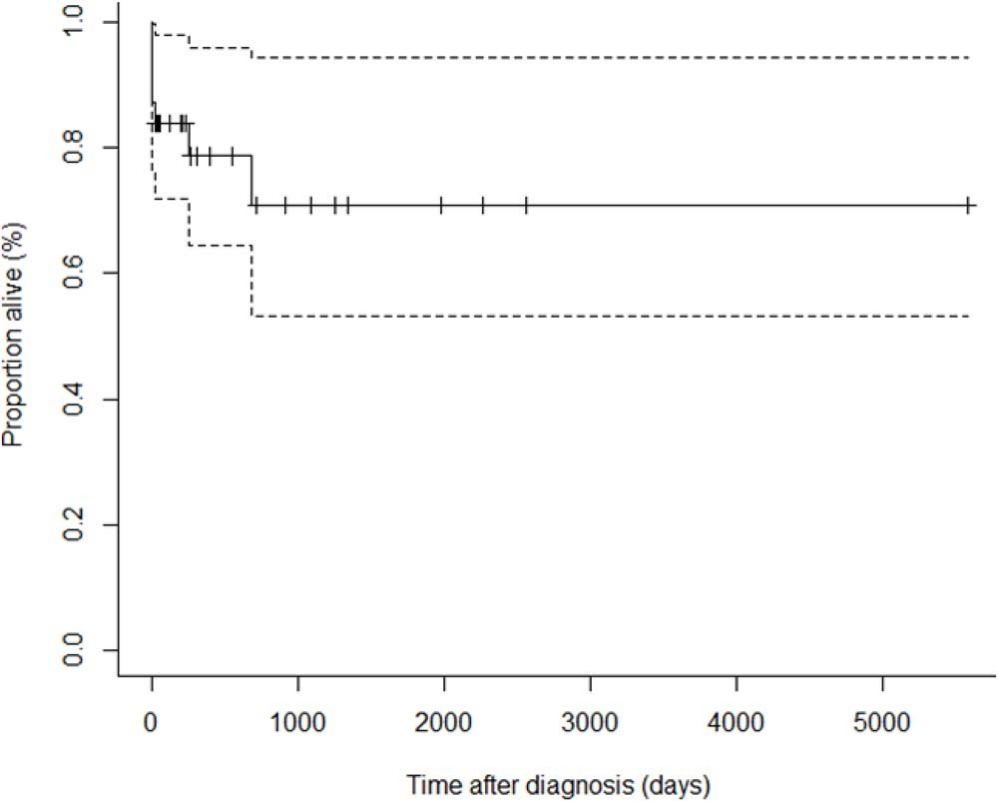
Kaplan–Meier estimates of survival for cats with primary immune thrombocytopenia (*n* = 31). Censored indicates that the event (death or euthanasia associated with immune thrombocytopenia) did not occur in the time the cat had been followed up. The dashed lines represent 95% confidence intervals around each survival estimate.

The median survival was not reached, as illustrated in Figure 2. The median follow-up time was 315 days (95% CI, 216-1252 days). There were 11 deaths in total, 7 of which were euthanized for reasons associated with primary ITP at a median of 7 days (range: 2-679 days). Four of these cats did not survive to discharge and were euthanized due to persistent thrombocytopenia and anemia. The other 3 survived to discharge, 2 were euthanized as they did not achieve medical control and had persistent thrombocytopenia, and one was euthanized at 679 days after a second relapse. The remaining cats died of reasons unassociated with primary ITP (*n* = 4), including euthanasia attributable to multiple chronic health issues (*n* = 2), aortic thromboembolism (*n* = 1), and an unspecified reason (*n* = 1).

### Efficacy of immunosuppressive protocols

Corticosteroids as single agent were compared with combination immunosuppressive treatment against clinical outcomes, the results of which are outlined in Tables 1 and 2. The use of combination treatment was significantly associated with a longer hospitalization duration in days compared with cats administered corticosteroids alone (*P* = .001), with a predicted mean of 8.4 days (±SE 0.7) and 4.4 days (±SE 0.8), respectively. Corticosteroids as a single agent were compared with corticosteroids and cyclosporine treatment against clinical outcomes (Tables 1 and 2). The use of corticosteroids and cyclosporine were significantly associated with a longer hospitalization duration in days compared with cats administered corticosteroids alone (*P* = .005), with a predicted mean of 8.4 days (±SE 0.9) and 4.4 days (±SE 0.9), respectively.

**Table 1:** Logistic regression model results comparing cats with primary immune thrombocytopenia treated with corticosteroids alone against combination immunosuppressive treatment or corticosteroid and cyclosporine treatment on measured clinical outcomes with a binomial distribution.

	**Clinical outcomes**	**Crude OR**	**95% CI**	***P*-value**
**Corticosteroids alone vs combination immunosuppressive treatment**	Occurrence of medical control	1.50	0.251-8.98	.657
	Occurrence of relapse	0.242	0.040-1.33	.104
	Occurrence of remission	1.17	0.208-6.56	.861
	Occurrence of death associated with primary ITP	1.33	0.113-15.7	.819
	Survival to discharge	0.417	0.038-4.53	.450
**Corticosteroids alone vs corticosteroids and cyclosporine**	Occurrence of medical control	0.90	0.143-5.65	.910
	Occurrence of relapse	0.33	0.051-2.18	.242
	Occurrence of remission	1.17	0.168-8.09	.876
	Occurrence of death associated with primary ITP	1.33	0.113-15.7	.819
	Survival to discharge	0.25	0.022-2.82	.231

The crude odds ratio (OR) and 95% confidence interval (CI) are displayed.

Abbreviations: ITP = immune thrombocytopenia; PCV = packed cell volume.

**Table 2:** Linear model results to compare cats with primary immune thrombocytopenia that received corticosteroids alone against combination immunosuppressive treatment or corticosteroid and cyclosporine treatment on measured clinical outcomes with a continuous distribution.

	**Clinical outcomes**	**Constant**	**Standard error**	***P*-value **
**Corticosteroids alone vs combination immunosuppressive treatment**	Days to medical control	6.71	9.82	.202
	Days to remission	308	67.2	.717
	Hospitalization duration	8.41	0.70	**.001**
**Corticosteroids alone vs corticosteroids and cyclosporine**	Days to medical control	26.7	13.6	.395
	Days to remission	263	121	.637
	Hospitalization duration	4.42	0.862	**.005**

Statistically significant differences at *P* < .05 are bolded. The constant and standard error are displayed.

### Prognostic indicators

Eight variables, including age, sex, breed, bodyweight, initial platelet count and PCV, blood product administration, and BUN concentration above reference range, were statistically analyzed as possible prognostic indicators for cats with primary ITP, with the results as outlined in Tables 3 and 4. Increasing initial bodyweight was significantly associated with an increased likelihood of achieving medical control (*P* = .007).

**Table 3:** Logistic regression model results to evaluate possible prognostic factors on measured clinical outcomes with a binomial distribution in cats with primary immune thrombocytopenia.

**Variable**	**Clinical outcomes**	**Crude OR**	**95% CI**	***P*-value **
**Age**	Occurrence of medical control	1.00	0.985-1.03	.638
	Occurrence of relapse	1.714	0.340-8.68	.512
	Occurrence of remission	0.985	0.964-1.01	.162
	Occurrence of death associated with primary ITP	0.998	0.971-1.03	.880
	Survival to discharge	1.01	0.983-1.03	.529
**Sex**	Occurrence of medical control	2.17	0.334-14.1	.407
	Occurrence of relapse	0.875	0.176-4.34	.870
	Occurrence of remission	0.889	0.165-4.78	.891
	Occurrence of death associated with primary ITP	0.75	0.063-8.83	.819
	Survival to discharge	3.23	0.297-35.1	.305
**Breed**	Occurrence of medical control	0.636	0.098-4.14	.631
	Occurrence of relapse	2.67	0.490-14.5	.245
	Occurrence of remission	1.48	0.266-8.27	.652
	Occurrence of death associated with primary ITP	7.50	0.450-123	.131
	Survival to discharge	1.45	0.177-11.9	.728
**Bodyweight**	Occurrence of medical control	3.81	1.18-12.33	**.007**
	Occurrence of relapse	1.13	0.216-5.86	.889
	Occurrence of remission	0.787	0.368-1.68	.515
	Occurrence of death associated with primary ITP	0.218	0.031-1.56	.081
	Survival to discharge	1.72	0.607-4.87	.286
**Initial platelet count**	Occurrence of medical control	1.14	0.945-1.38	.067
	Occurrence of relapse	1.02	0.950-1.10	.524
	Occurrence of remission	0.992	0.917-1.07	.829
	Occurrence of death associated with primary ITP	1.01	0.910-1.13	.794
	Survival to discharge	1.09	0.911-1.30	.248
**Initial PCV**	Occurrence of medical control	0.959	0.847-1.09	.514
	Occurrence of relapse	1.10	0.960-1.27	.139
	Occurrence of remission	1.06	0.920-1.22	.424
	Occurrence of death associated with primary ITP	1.06	0.870-1.29	.581
	Survival to discharge	0.956	0.828-1.10	.546
**Transfusion occurrence**	Occurrence of medical control	1.67	0.270-10.1	.579
	Occurrence of relapse	0.291	0.050-1.66	.158
	Occurrence of remission	0.313	0.050-1.97	.206
	Occurrence of death associated with primary ITP	0.750	0.064-8.83	.819
	Survival to discharge	0.455	0.042-4.98	.499
**BUN concentration above reference range**	Occurrence of medical control	0.381	0.05-2.83	.359
	Occurrence of relapse	0.444	0.04-5.01	.493
	Occurrence of remission	0.667	0.06-7.64	.739
	Occurrence of death associated with primary ITP	0.21	0.040-2.89	.152
	Survival to discharge	0.682	0.06-8.00	.766

Statistically significant differences at *P* < .05 are bolded. The crude odds ratio (OR) and 95% confidence interval (CI) are displayed.

Abbreviations: BUN = blood urea nitrogen; ITP = immune thrombocytopenia; PCV = packed cell volume.

**Table 4:** Linear model results to evaluate possible prognostic factors on measured clinical outcomes with a continuous distribution in cats with primary immune thrombocytopenia.

**Variable**	**Clinical outcomes**	**Constant**	**Standard error**	***P*-value **
**Age**	Days to medical control	27.3	12.3	0.221
	Days to remission	314	76.3	0.704
	Hospitalization duration	5.75	1.04	0.227
**Sex**	Days to medical control	0.75	0.10	0.428
	Days to remission	281	78.5	0.839
	Hospitalization duration	7.44	0.86	0.250
**Breed**	Days to medical control	5.64	11.2	0.265
	Days to remission	381	48.4	0.061
	Hospitalization duration	7.46	0.96	0.335
**Bodyweight**	Days to medical control	6.07	0.85	.301
	Days to remission	368	96.1	.484
	Hospitalization duration	6.07	0.85	.301
**Initial platelet count**	Days to medical control	17.7	11.1	.738
	Days to remission	309	106	.865
	Hospitalization duration	7.35	0.91	.357
**Initial PCV**	Days to medical control	3.93	3.46	.318
	Days to remission	420	128	.401
	Hospitalization duration	9.36	1.46	.066
**Transfusion occurrence**	Days to medical control	30.2	12.3	.142
	Days to remission	246	81.6	.453
	Hospitalization duration	5.40	1.08	.112
**BUN concentration above reference range**	Days to medical control	16.3	8.51	.731
	Days to remission	265	51.1	.258
	Hospitalization duration	6.87	0.74	.743

The constant and standard error are displayed. The clinical outcome days to medical control was analyzed after log transformation.

Abbreviations: BUN = blood urea nitrogen; PCV = packed cell volume.

## Discussion

This large retrospective study of cats with primary ITP examines treatment outcomes, prognosis, and prognostic indicators. Cats that received combination immunosuppressive therapy, specifically corticosteroids and cyclosporine, had a longer duration of hospitalization compared with cats that received corticosteroids only. The study cohort was relatively young and had a fair short-term prognosis with 87% surviving to discharge. There was a low remission rate of 32% and a high combined relapse and recurrence rate of 44%. Of the cats that relapsed once, 50% had a second relapse.

Cats that received combination immunosuppressive therapy, specifically corticosteroids and cyclosporine, had a longer duration of hospitalization compared to corticosteroids as a single agent. The cats had a predicted mean duration of hospitalization of 8 and 4 days, respectively. In comparison, dogs treated with corticosteroids alone have a median hospitalization of 8 days.^22^ Of the cats that received corticosteroid and cyclosporine therapy, 10 (83%) were administered corticosteroids as a single agent on the day of presentation. The difference between the 2 treatment groups might be related to factors influencing the decision to commence additional immunosuppressives, including inadequate platelet response, a decline in PCV, and the development of additional clinical signs of thrombocytopenia. Given that most of these cats were only administered corticosteroids initially, these cats might have needed more time to achieve platelet response or were refractory to initial treatment, therefore requiring additional immunosuppressants and more time in hospital. The response time for achieving an increase in platelet count in cats after corticosteroid administration is unclear. The time for the platelet count in dogs to rise above 40 000/μL after a 1.5-2 mg/kg twice a day dosage of prednisolone is 6.5 days.^23^ The consensus on the indications for second agent commencement in cats include severe hemorrhage, a lack of response 5-7 days after corticosteroid treatment, severe adverse effects of corticosteroids, and the occurrence of a relapse.^11^ Clinicians could consider administering cyclosporine if there is an inadequate platelet response 4 days into hospitalization, which was the predicted mean hospitalization duration of cats receiving corticosteroids as a single agent. No other significant differences were found when comparing cats that received combination immunosuppressive treatment to corticosteroids as a single agent. Similarly, there were no other significant differences detected between cats receiving the specific combination of corticosteroids and cyclosporine with corticosteroids as a single agent on other measured clinical outcomes. In dogs, there are no differences between these 2 treatment groups on survival to discharge and relapse.^12^ There was an insufficient number of cats that received other treatment combinations for statistical analysis to be performed on these groups.

The short-term prognosis of cats with primary ITP in this cohort was fair, with 87% surviving to discharge. All 7 deaths associated with primary ITP were from euthanasia, and 6 of these cats reportedly did not achieve medical control. The remaining cat that reached medical control was euthanized at the second relapse. The median survival time was not reached, with most censoring associated with loss of follow-up, which occurred at varying intervals. However, no deaths or euthanasia justifiably attributable to ITP were recorded after 679 days. In 2 previous case reports of cats with primary ITP, 2/8 (25%) cats were euthanized because of pulmonary hemorrhage or suspected thromboembolism.^6^^,^^7^ Dogs have a variable but fair short-term prognosis, with 84%-97% of dogs surviving to discharge.^13–17^ In this study, remission was achieved in 32% of cats, where they were completely weaned of immunosuppressive agents. Remission is not reported in cats with ITP. One cat that reached remission then had a recurrence of primary ITP. Of the cats within this study, 81% reached medical control and 40% of these cats had at least 1 relapse during immunosuppressive agent tapering, with an overall relapse and recurrence rate of 44%. Relapse rates are variable in dogs, with recent reports between 9% and 31% and older reports between 26% and 47%.^13–15^^,^^17^^,^^18^ Of the cats that relapsed once, 50% had a further relapse, which is similar to previous reports in dogs.^13^ Therefore, it is recommended for clinicians and owners to continue closely monitoring cats after their first relapse. Nevertheless, immunosuppressants can still be tapered in cats that have relapsed after stabilization, as one cat with multiple relapses achieved remission.

Cats with primary ITP in this study were relatively young, with a median age of 4 years and 9 months. Over half of the cohort was comprised of pedigree breeds. A large proportion were British Shorthair cats, with all but one identified from a hospital in the United Kingdom. Cats with a higher bodyweight were also more likely to reach medical control. The increased likelihood in achieving medical control might be attributable to cats with higher bodyweights receiving greater immunosuppressive dosages relative to their lean body mass. The negative prognostic indicators in dogs, including melena, a lower PCV, and a BUN concentration above the reference range, could all potentially be associated with gastrointestinal hemorrhage or not associated.^14^^,^^19^^,^^20^^,^^24^ Melena was not a common clinical sign in this study cohort, and was not statistically analyzed as a prognostic indicator. Concurrent renal disease as a cause of BUN concentration above the reference range cannot be excluded in these cats, as a urinalysis was not part of the inclusion criteria and was often not performed during initial diagnostic testing because of risks of hemorrhage with cystocentesis in cats with ITP.

Evans syndrome might have been under- or overidentified in this cohort. Immune-mediated hemolytic anemia was assessed according to the American College of Veterinary Internal Medicine consensus statement.^25^ Cats with results supportive of Evans syndrome were excluded and not further analyzed because of the small sample size. The widespread geographical distribution of cats included leads to differing prevalence rates of infectious diseases. Hemotropic mycoplasmas were the most common infectious disease tested as a cause of anemia.^26^ Doxycycline was initially administered empirically to multiple cats, with only a few tested for hemotropic mycoplasmas. Cats not tested for hemotropic mycoplasmas were included given that their association with thrombocytopenia is equivocal and because of the relatively low prevalence of the most pathogenic *Mycoplasma haemofelis*.^26–29^ Detection of platelet-specific autoantibodies with flow cytometric assay can provide immunologic evidence, but was not an inclusion criterion because of its limited accessibility.^4^^,^^25^

A limitation of this study is that cats with other causes of thrombocytopenia could have been inadvertently included. All cats were FIV and FeLV tested, with most using POC testing. There is a possibility of false negatives, for example, if testing was performed during the initial stage of infection.^30^ One cat faintly positive on FeLV antigen testing had a negative polymerase chain reaction test, suggesting an unclear, but likely negative status.^30^^,^^31^ Disseminated intravascular coagulation is diagnostically challenging because of the lack of standardized diagnostic criteria in cats and the dynamic nature of the condition.^32^ A full coagulation panel was not performed in the included cats, therefore disseminated intravascular coagulation, neoplasia, feline infectious peritonitis, and liver failure cannot be completely excluded.^32^^,^^33^ A primary bone marrow disorder cannot be excluded in cats that did not receive a bone marrow examination. However, these cats had an absence of abnormal peripheral blood findings besides thrombocytopenia and anemia. The cat with possible megakaryocytic hypoplasia was included as megakaryocytic hyperplasia is not pathognomonic for primary ITP. While dogs with primary ITP more commonly have megakaryocytic hyperplasia, megakaryocytic hypoplasia has been reported.^12^ Hyperthyroidism was concurrently diagnosed in 1 cat. This cat was included as it was not previously receiving antithyroid drugs and because Graves’ disease, an autoimmune thyroid disease that can cause concomitant ITP in humans, is not described in cats.^34^

The multicenter retrospective study design inherently carries several limitations. Numerous hospitals were contacted to maximize case recruitment and a clinician from each hospital extracted data for identified cats. Bias was reduced through the eligibility criteria and the request for unambiguous objective case details. Cats identified were checked against the study’s eligibility criteria by 2 authors (M.Z./L.B.) to reduce the effect of selection bias. The effect of this bias, if present, is difficult to estimate. The nonsignificant findings could represent a type II statistical error because the study likely was underpowered attributable to a small sample size. Some data was lacking, which could have led to an under or overestimation of the odds ratio. To reduce spurious platelet counts from different in-house hematology analyzers and platelet clumping, manual platelet counts and external pathology results were preferentially recorded.^35^ Although performing a manual platelet count reduces misclassification of pseudothrombocytopenic cats, inaccuracies with the number of platelets might persist. A prospective study would more effectively address platelet aggregation in cats, through the standardization of sample handling, preparation, and platelet counting. There was no standardization to follow-up; therefore, data concerning long-term clinical outcomes should be interpreted with caution. Management of the cats was also at the discretion of the attending clinician, which led to variation in immunosuppressants, dosages, and tapering protocols. Regarding survival analysis, all deaths within the cohort associated with primary ITP were caused by euthanasia. The survival time of these cats is likely biased given that the timing of euthanasia is at the discretion of the owner and can be influenced by other factors besides disease progression.^36^ A prospective multicenter study of cats with primary ITP with standardized testing and treatment protocols is recommended to investigate treatment outcomes and prognostic indicators further; however, this would be challenging, given the rare incidence of primary ITP in cats.

In conclusion, primary ITP in cats is rare and carries a fair short-term prognosis with a survival to discharge rate of 87%, although the median survival was not reached. Cats receiving combination immunosuppressive treatment, specifically corticosteroids and cyclosporine, had a longer hospitalization than those treated with corticosteroids alone. Most cats on a combination immunosuppressive treatment received corticosteroids as a single agent initially. Cyclosporine could be started earlier at 4 days in cats with an inadequate response to corticosteroids, which was the predicted mean hospitalization duration of cats receiving corticosteroids alone. There was a low remission rate of 32%, with a high combined relapse and recurrence rate of 44%. These cats had relatively frequent repeat relapses, warranting closer monitoring.

## Off-label antimicrobial declaration

The authors declare no off-label use of antimicrobials.

## Institutional animal care and use committee or other approval declaration

Approved by the Ethical and Administrative Committee at the Department of Veterinary Clinical Sciences, University of Copenhagen, reference number 2023-31.

## Abbreviations

BUN: blood urea nitrogen
FIV: feline immunodeficiency virus
FeLV: feline leukemia virus
ITP: immune thrombocytopenia
POC: point-of-care
