# Correction to: Treatment outcomes and prognostic indicators of primary immune thrombocytopenia in 31 cats: a multicenter retrospective study (2000-2023)

**DOI:** 10.1093/jvimsj/aalag106

**Published:** 2026-05-06

This is a correction to: Mulan Zhong, Evelyn Hall, Barbara Glanemann, Susan H S Jih, Rachel M Korman, Rebecca Langhorn, Jana Leshinsky, Amy E Lingard, Elodie Roels, Martine van Boeijen, Lara A Boland, Treatment outcomes and prognostic indicators of primary immune thrombocytopenia in 31 cats: a multicenter retrospective study (2000-2023), *Journal of Veterinary Internal Medicine*, Volume 40, Issue 1, January–February 2026, aalaf038, https://doi.org/10.1093/jvimsj/aalaf038

In the originally published version of the paper, Figure 2 “Kaplan–Meier estimates of survival for cats with primary immune thrombocytopenia (n = 31)” had two images published but should have only had a single image. Figure 2 has been corrected in the article to read:


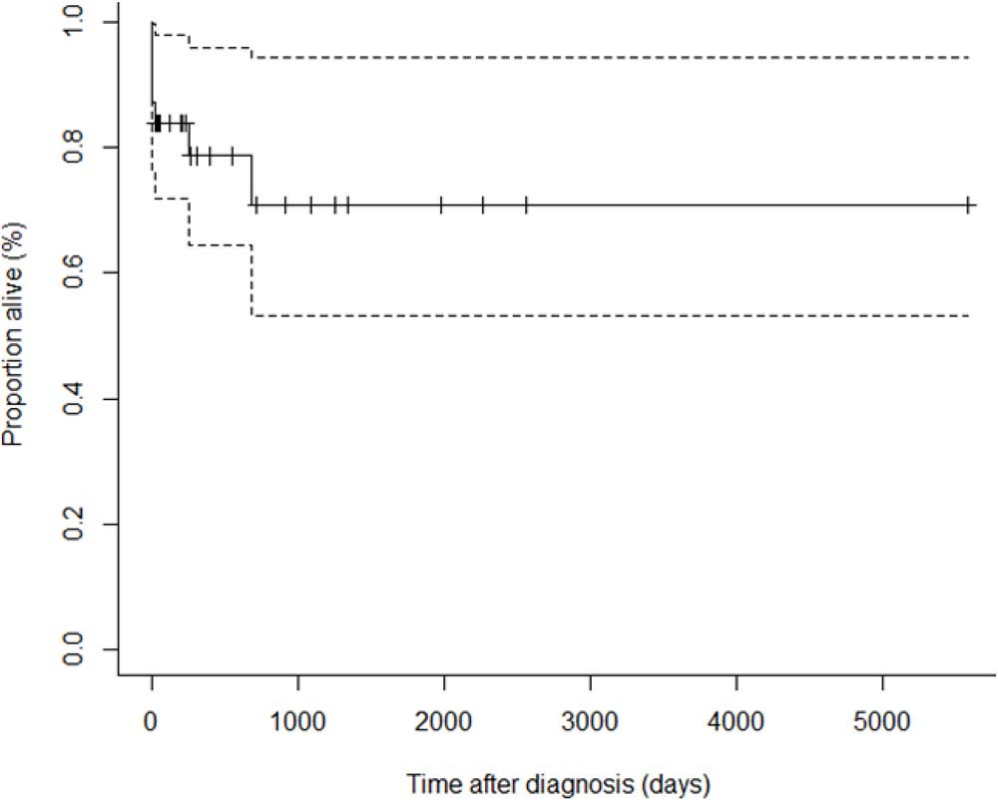


instead of:


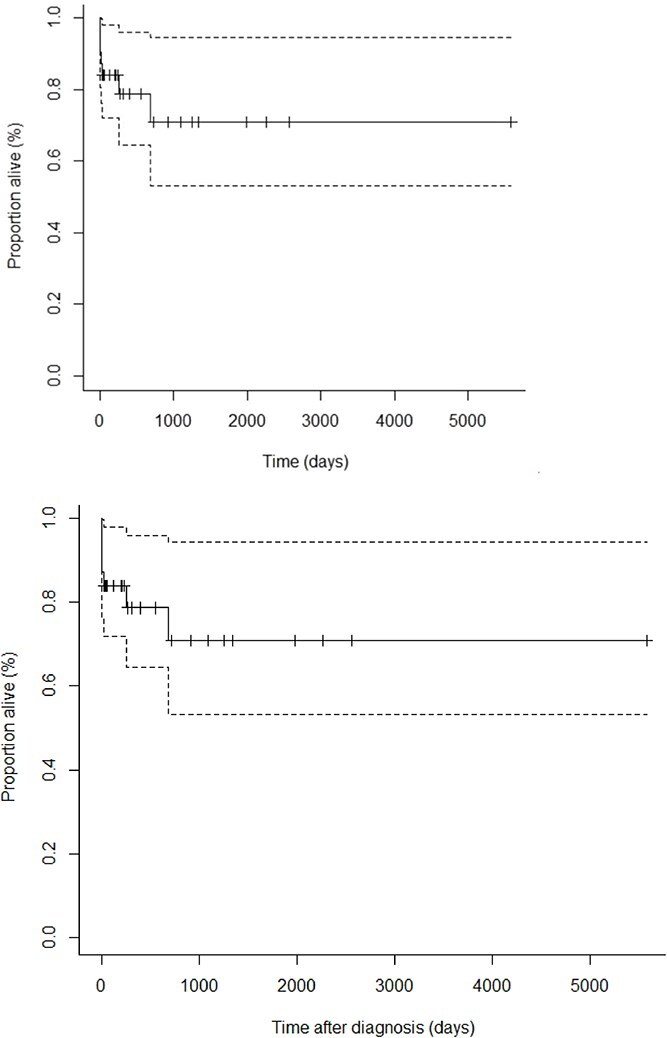


We apologise for the error.

